# Dual role of pericyte α6β1-integrin in tumour blood vessels

**DOI:** 10.1242/jcs.197848

**Published:** 2017-05-01

**Authors:** Louise E. Reynolds, Gabriela D'Amico, Tanguy Lechertier, Alexandros Papachristodoulou, José M. Muñoz-Félix, Adèle De Arcangelis, Marianne Baker, Bryan Serrels, Kairbaan M. Hodivala-Dilke

**Affiliations:** 1 Adhesion and Angiogenesis Laboratory, Centre for Tumour Biology, Barts Cancer Institute - A CRUK Centre of Excellence, Queen Mary University of London, Charterhouse Square, London EC1M 6BQ, UK; 2 Laboratory for Molecular Neuro-Oncology, Dept. of Neurology, University Hospital Zurich, Frauenklinikstrasse 26, Zurich CH-8091, Switzerland; 3 IGBMC, UMR 7104, INSERM U964, Université de Strasbourg, BP. 10142, 1, Rue Laurent Fries, Illkirch Cedex 67404, France; 4 Cancer Research UK Edinburgh Centre, University of Edinburgh, Crewe Road South, Edinburgh EH4 2XR, UK

**Keywords:** Integrin, Pericyte, Tumour growth, Angiogenesis

## Abstract

The α6β1-integrin is a major laminin receptor, and formation of a laminin-rich basement membrane is a key feature in tumour blood vessel stabilisation and pericyte recruitment, processes that are important in the growth and maturation of tumour blood vessels. However, the role of pericyte α6β1-integrin in angiogenesis is largely unknown. We developed mice where the α6-integrin subunit is deleted in pericytes and examined tumour angiogenesis and growth. These mice had: (1) reduced pericyte coverage of tumour blood vessels; (2) reduced tumour blood vessel stability; (3) increased blood vessel diameter; (4) enhanced blood vessel leakiness, and (5) abnormal blood vessel basement membrane architecture. Surprisingly, tumour growth, blood vessel density and metastasis were not altered. Analysis of retinas revealed that deletion of pericyte α6-integrin did not affect physiological angiogenesis. At the molecular level, we provide evidence that pericyte α6-integrin controls PDGFRβ expression and AKT–mTOR signalling. Taken together, we show that pericyte α6β1-integrin regulates tumour blood vessels by both controlling PDGFRβ and basement membrane architecture. These data establish a novel dual role for pericyte α6-integrin as modulating the blood vessel phenotype during pathological angiogenesis.

## Abstract (precis)

**Summary:** Mice with pericyte-specific deletion of α6-integrin had reduced pericyte blood vessel coverage, increased vessel leakiness and abnormal blood vessel architecture. α6-integrin also controls pericyte responses to PDGF-BB.

## INTRODUCTION

Blood vessels comprise endothelial cells supported by mural cells, also known as pericytes. Although the role of integrins in endothelial biology has been studied extensively (Avraamides et al., 2008; Germain et al., 2010), almost nothing is known about the role of pericyte integrins, including α6-integrin (Garmy-Susini et al., 2005; Liu and Leask, 2012). Integrins are transmembrane cell surface receptors that mediate cell–cell and cell–extracellular matrix (ECM) interactions. One of the major components of the vascular basement membrane is laminin whose predominant adhesive receptors include the integrins α6β1 and α6β4 (Durbeej, 2010). Targeting endothelial integrins has proved to be relatively beneficial in treating cancer in preclinical studies (Yamada et al., 2006; Tabatabai et al., 2010) but has had limited success in clinical trials (Patel et al., 2001), therefore new targets, including pericytes are currently being examined.

Tumour blood vessels have many structural abnormalities including decreased endothelial barrier function, reduced pericyte recruitment and poor basement membrane organisation when compared with normal quiescent vessels (Armulik et al., 2005). Studies have shown that pericyte recruitment and investment to blood vessels stimulates endothelial cell basement membrane (BM) deposition and organisation *in vitro* (Stratman et al., 2009). This is mediated mainly by secretion of endothelial platelet-derived growth factor (PDGF)-BB, attracting PDGF receptor β (PDGFRβ)-positive pericytes, which adhere to the BM surrounding endothelial cells (Stratman et al., 2010; Abramsson et al., 2003). *In vivo*, mice lacking PDGF-BB–PDGFRβ signalling fail to adequately recruit pericytes to newly formed blood vessels, resulting in severe perturbation of blood vessel stabilisation and maturation (Hellberg et al., 2010). Furthermore, interference with PDGF-BB–PDGFRβ signalling results in disruption of already established endothelial–pericyte associations and vessel destabilisation during retinal development (Benjamin et al., 1998). Whether pericyte α6-integrin might regulate tumour vessel stability was hitherto unknown.

In the present study, we examined the role of pericyte α6-integrin on tumour blood vessel function using a genetic ablation approach. Surprisingly, loss of pericyte α6-integrin did not affect tumour growth, angiogenesis or metastasis but did cause a decrease in pericyte association with tumour blood vessels and poor basement membrane organisation, with an associated increase in vessel leakage and instability. At the molecular level, we demonstrate a novel mechanism by which pericyte α6-integrin reduces PDGFRβ expression on pericytes and therefore diminishes responses to PDGF-BB. This, in turn, is the likely mechanism for aberrant pericyte investment of tumour blood vessels. Taken together, our data suggest that pericyte α6β1-integrin plays a dual role in regulating PDGFRβ expression and BM organisation that likely increases vessel leakage and instability.

## RESULTS

### Generation and characterisation of *pdgfrβcre+;α6fl/fl* mice

We have generated a new mouse model that enables us to study deletion of α6-integrin in pericytes. We bred α6-integrin floxed mice (denoted *α6fl/fl*) (Bouvard et al., 2012; Germain et al., 2010) with mice expressing Cre-recombinase under the control of the PDGFRβ promoter (denoted *pdgfrβcre+*) (Foo et al., 2006), to generate *pdgfrβcre-;α6fl/fl* and *pdgfrβcre+;α6fl/fl* mice. Mice were born to *pdgfrβcre+;α6fl/fl*×*pdgfrβcre-;α6fl/fl* crosses at normal Mendelian ratios and male:female ratios with no obvious adverse phenotype (Fig. S1A–C). Mice were genotyped by PCR analysis (Fig. S1D). Histological analysis of H&E-stained sections of lung, heart, liver and spleen from *pdgfrβcre-;α6fl/fl* and *pdgfrβcre+;α6fl/fl* adult mice showed no apparent tissue defects (Fig. S1E). Furthermore, no apparent vascular abnormalities were observed in these tissues (Fig. S1F) or in the developing retina (Fig. S1G), suggesting that loss of PDGFRβ-driven α6-integrin had no apparent effect on physiological angiogenesis. Finally, to confirm pericyte-specific Cre expression in our mouse model, we crossed *pdgfrβcre-* and *pdgfrβcre+* mice with the *mTmG* reporter mouse, which expresses membrane-targeted tandem dimer Tomato (mT; red) prior to Cre-mediated excision and membrane-targeted green fluorescent protein (mG; green) after excision (Muzumdar et al., 2007). Analysis of tumour blood vessels from *pdgfrβcre-;mTmG* and *pdgfrβcre+;mTmG* mice showed that although Tomato (mT) expression was observed in blood vessels in both *pdgfrβcre-;mTmG* and *pdgfrβcre+;mTmG mice*, GFP (mG) expression, was present in mouse tissue only after Cre excision, and only observed in pericytes in *pdgfrβcre+;mTmG* mice (Fig. S2A,B). As expected, α6-integrin is expressed on tumour endothelial cells, shown by co-expression of α6-integrin and the endothelial cell marker CD31 (Fig. S2C), suggesting that the deletion of α6-integrin *in vivo* was restricted to PDGFRβ-positive pericytes.

### α6-integrin deficiency on pericytes impairs tumour blood vessel stabilisation

**Fig. 1.**
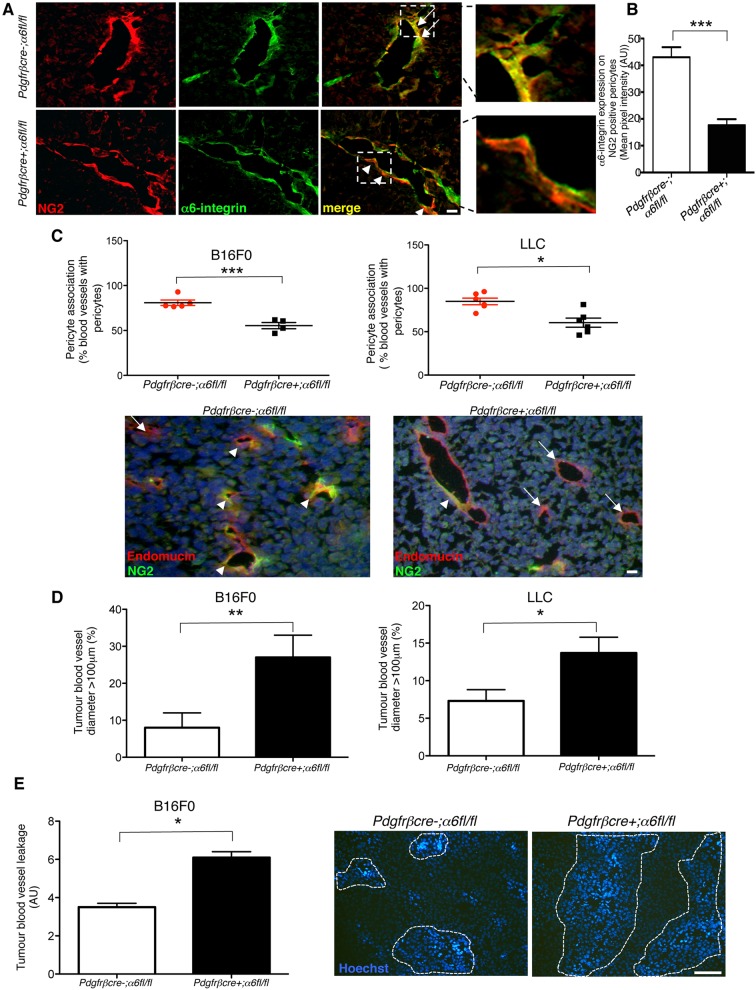
**Reduced pericyte investment and increased vessel leakage in *pdgfrβcre+;α6fl/fl* mice.** (A) B16F0 tumour sections from *pdgfrβcre-;α6fl/fl* and *pdgfrβcre+;α6fl/fl* mice were double-immunostained for NG2 (red) and α6-integrin (green) expression to determine *in vivo* expression of α6-integrin in pericytes. α6-integrin was observed in NG2-positive pericytes in tumour blood vessels from *pdgfrβcre-;α6fl/fl* mice. In contrast, α6-integrin expression was significantly reduced in pericytes in tumour blood vessels from *pdgfrβcre+;α6fl/fl* mice. Magnified regions show α6-integrin-positive pericytes (to give a yellow signal) on *pdgfrβcre-;α6fl/fl* blood vessels but α6-integrin-negative pericytes on *pdgfrβcre+;α6fl/fl* blood vessels. Arrows, α6 and NG2 co-expression; arrowheads, NG2 expression alone. (B) Quantification of α6-integrin expression in NG2-positive pericytes from tumour blood vessels in *pdgfrβcre-;α6fl/fl* in *pdgfrβcre+;α6fl/fl* mice. Bar chart represents mean+s.e.m. pixel intensity of α6-integrin expression in NG2-positive cells; *n*=9–10 tumours per genotype. (C) Pericyte association with blood vessels. The percentage of blood vessels with associated NG2-positive pericytes in B16F0 and LLC tumours grown in *pdgfrβcre+;α6fl/fl* mice was reduced significantly compared with *pdgfrβcre-;α6fl/fl* mice. Scatter graphs represent the mean+s.e.m. percentage blood vessels that are NG2-positive in B16F0 and LLC tumours; *n*=4–6 mice/group. Representative images of tumour sections stained with the pericyte marker NG2 (green) and for endomucin (red). Arrows, endomucin-positive staining; arrowheads, NG2-positive staining. (D) Blood vessel diameter. The frequency of vessels with a diameter ≥100 µm was greater in both B16F0 and LLC tumours grown in *pdgfrβcre+;α6fl/fl* mice when compared with *pdgfrβcre-;α6fl/fl* mice. Bar charts represent the mean+s.e.m. percentage of blood vessels ≥100 µm diameter. (E) Blood vessel leakage. Mice were injected via the tail vein with Hoechst 33258 dye and tumour sections were analysed for blood vessel leakage by measuring the numbers of tumour cells that had taken up Hoechst. Blood vessels in the tumours grown in *pdgfrβcre+;α6fl/fl* mice showed significantly more leakage than blood vessels in tumours grown in *pdgfrβcre-;α6fl/fl* mice. Bar chart shows relative mean+s.e.m. leakage; *n*=6 tumours/genotype. Dotted lines, Hoechst leakage. *P<0.05, **P<0.005, ***P<0.0009. Scale bars: 50 µm (A), 100 µm (C), 200 µm (E).

Very few studies have examined the role of pericyte integrins in tumour blood vessels *in vivo* (Garmy-Susini et al., 2005; Turner et al., 2014; Liu and Leask, 2012)*.* To assess whether α6-integrin is required for pericyte investment on blood vessels during tumour angiogenesis, *pdgfrβcre-;α6fl/fl* and *pdgfrβcre+;α6fl/fl* mice were injected with either syngeneic B16F0 melanoma or Lewis lung carcinoma (LLC) tumour cells. We first established loss of α6-integrin expression in pericytes in tumour blood vessels from *pdgfrβcre+;α6fl/fl* mice. Tumour sections from *pdgfrβcre-;α6fl/fl* and *pdgfrβcre+;α6fl/fl* were double stained for the NG2 proteoglycan (a pericyte marker) and α6-integrin. Although α6-integrin was expressed in NG2-positive pericytes in tumour blood vessels in *pdgfrβcre-;α6fl/fl* control mice, α6-integrin was undetectable in pericytes from *pdgfrβcre+;α6fl/fl* mice (Fig. 1A) confirming that α6-integrin expression is reduced on pericytes in tumours grown in *pdgfrβcre+;α6fl/fl* mice. Quantification revealed a significant reduction in α6-integrin expression in NG2-positive pericytes in tumour blood vessels from *pdgfrβcre+;α6fl/fl* mice (Fig. 1B). Having confirmed deletion of α6-integrin in pericytes in tumour blood vessels from *pdgfrβcre+;α6fl/fl* mice *in vivo*, we asked whether PDGFRβ-driven α6-integrin deletion could affect pericyte association with tumour blood vessels. A significant reduction in the number of pericytes associated with blood vessels in B16F0 and LLC tumours grown in *pdgfrβcre+;α6fl/fl* mice was observed (Fig. 1C). Thus, the absence of pericyte α6-integrin corresponds with a reduction in pericyte association with blood vessels.

### Blood vessel leakage is enhanced in tumours from *pdgfrβcre+; α6fl/fl* mice

We next examined other possible biological consequences of the loss of pericyte α6-integrin. We found that reduced pericyte investment correlated with a higher frequency of blood vessels with a large diameter (>100 µm) in tumour blood vessels from *pdgfrβcre+;α6fl/fl* mice (Fig. 1D). A known result of increased blood vessel diameter is poor functionality of blood vessels (Huang et al., 2010). We therefore examined tumour blood vessel leakage by measuring the relative level of perivascular Hoechst 33258 after intravenous injection of Hoechst dye. Blood vessel leakage was calculated by counting the numbers of Hoechst-positive nuclei surrounding tumour blood vessels. Tumour blood vessels from *pdgfrβcre+;α6fl/fl* mice showed significantly more leakage of Hoechst dye than from *pdgfrβcre-;α6fl/fl* mouse tumours (Fig. 1E). Taken together, these data demonstrate that loss of pericyte α6-integrin is sufficient to reduce pericyte coverage of tumour blood vessels and increase vessel diameter and leakage.

### Blood vessel basement membrane organisation is altered in tumours grown in *pdgfrβcre+;α6fl/fl* mice

**Fig. 2.**
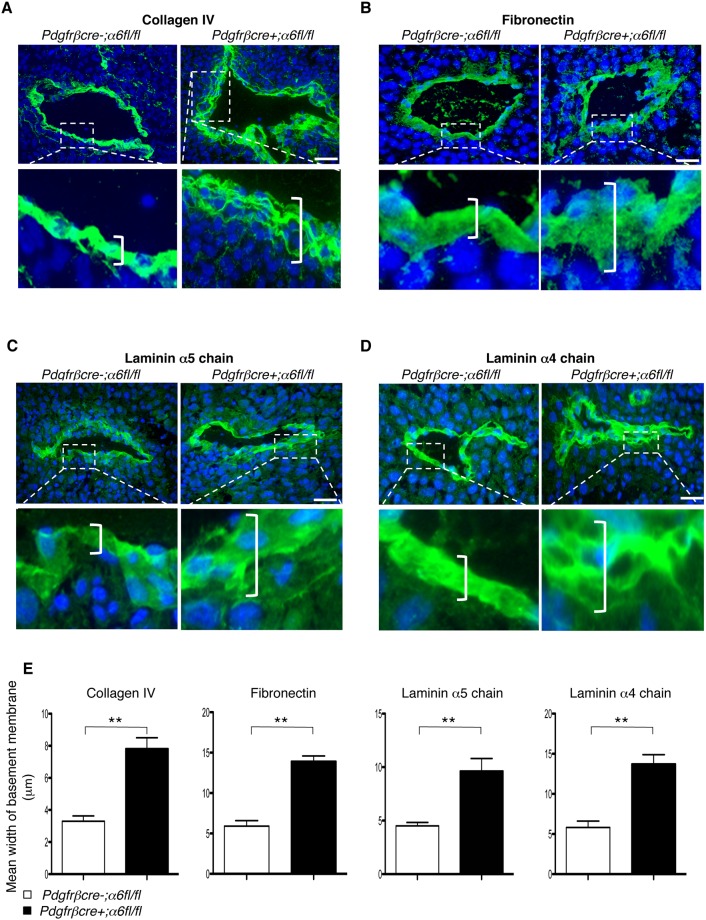
**Tumours grown in *pdgfrβcre+;α6fl/fl* mice have aberrant BM organisation around blood vessels.** LLC tumours were stained with antibodies to the ECM proteins (A) collagen IV, (B) fibronectin, (C) laminin α5 and (D) laminin α4 chains. For all matrices, disorganisation of BM with a ‘shoreline’ pattern was observed more frequently around blood vessels in tumours grown in *pdgfrβcre+;α6fl/fl* mice when compared with *pdgfrβcre-;α6fl/fl* mice. Boxes show magnified regions of BM. Brackets, identify representative BM widths. Scale bars: 50 µm. (E) The width of the BM surrounding blood vessels was analysed (mean+s.e.m.); *n*=8–10 sections/genotype. **P<0.005.

Given that extracellular matrix (ECM) deposition is a crucial step in the maturation of tumour blood vessels, and requires the presence of both pericytes and endothelial cells, we examined whether the distribution of various BM matrices correlated with the observed decrease in pericyte tumour blood vessel association in *pdgfrβcre+;α6fl/fl* mice. Concomitant with reduced vascular stabilisation and increased leakiness in the *pdgfrβcre+;α6fl/fl* tumour blood vessels, a higher frequency of disorganised BM around tumour blood vessels was seen in *pdgfrβcre+;α6fl/fl* mice. Specifically, immunofluorescence analysis of collagen IV (Fig. 2A), fibronectin (Fig. 2B), and laminin α5 (Fig. 2C) and α4 chains (Fig. 2D) in the BM around tumour blood vessels, revealed a ‘shorelining’ pattern of BM components (DiPersio et al., 1997) in *pdgfrβcre+;α6fl/fl* mice. Quantification confirmed that the BM was wider, with aberrant organisation, in blood vessels from *pdgfrβcre+;α6fl/fl* mice than BM from *pdgfrβcre-;α6fl/fl* mice (Fig. 2E). Adhesion assays using mouse primary pericytes showed, as expected, that the absence of α6-integrin significantly reduced the adherence to laminin 111 (Lm-1), but not fibronectin (Fig. S3A).

Since blood vessel stabilisation is a consequence not only of BM deposition but the association of supporting cells (i.e. pericytes), these results show that pericyte investment and the formation of a normal BM is affected by deletion of pericyte α6-integrin, and likely contributes to the abnormal stability and leakage of tumour blood vessels in *pdgfrβcre+;α6fl/fl* mice.

### Depletion of pericyte α6-integrin does not affect tumour growth or metastasis

**Fig. 3.**
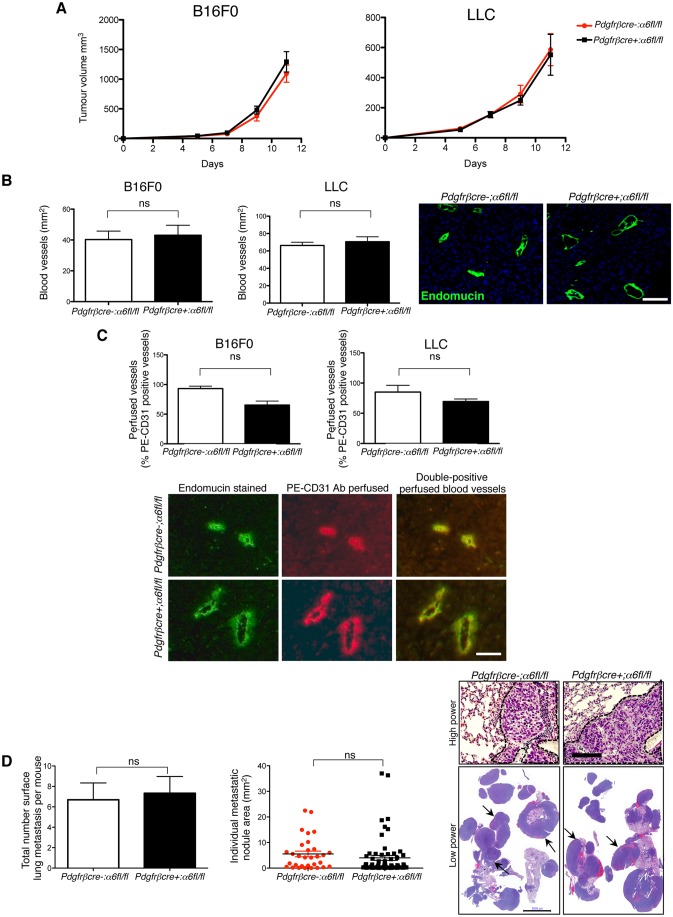
**Tumour growth, angiogenesis and metastasis are not affected in *pdgfrβcre+;α6fl/fl* mice.** (A) Subcutaneous B16F0 and LLC tumour growth was similar in both *pdgfrβcre+;α6fl/fl* mice and *pdgfrβcre-;α6fl/fl* mice. The bar charts represent mean±s.e.m. tumour volumes; *n*=20–30 mice/genotype. (B) Endomucin staining of midline sections of age- and size-matched B16F0 and LLC tumours showed no significant differences in blood vessel density between *pdgfrβcre+;α6fl/fl* mice and *pdgfrβcre-;α6fl/fl* mice. Blood vessel density is given as the number of blood vessels/mm^2^ for the midline tumour section. Representative images of endomucin-stained tumour blood vessels are shown. Bar chart represents mean+s.e.m. tumour blood vessel density of size-matched tumours; *n*=6 tumour sections/genotype, ns, no significant difference. (C) The number of perfused tumour blood vessels was assessed after tail vein injection of PE-conjugated anti-CD31 antibody before tumour excision in *pdgfrβcre+;α6fl/fl* mice and *pdgfrβcre-;α6fl/fl* control mice and comparing the numbers of CD31-positive vessels with numbers of endomucin-stained vessels. B16F0 and LLC tumours grown in *pdgfrβcre+;α6fl/fl* mice had a similar number of functional PE–CD31 endomucin-expressing tumour blood vessels compared to *pdgfrβcre-;α6fl/fl* littermate control mice. Bar charts represent the percentage of PE–CD31-perfused vessels over total number of endomucin-positive blood vessels for the midline tumour section from *pdgfrβcre+;α6fl/fl* mice and *pdgfrβcre-;α6fl/fl* mice+s.e.m.; *n*=10 tumour sections per genotype, ns, no significant difference. Representative images of endomucin-stained PE–CD31-positive perfused blood vessels from B16F0 tumours are shown. (D) Metastasis is not affected in *pdgfrβcre+;α6fl/fl* mice. Subcutaneous LLC tumours were resected when they reached ∼100 mm^3^. At 3 weeks post-resection, mice were killed and lungs removed to assess metastasis. Lungs were fixed and the number of surface metastases was counted. There were no significant differences in the total number of surface metastases between *pdgfrβcre+*;*α6fl/fl* mice and *pdgfrβcre-;α6fl/fl* mice (bar chart; mean+s.e.m.). Lungs were then sectioned and stained with H&E. Measurement of individual metastatic nodule areas showed no difference between genotypes (scatter graph). Representative high-power images of H&E-stained lung sections show areas of metastasis (dotted line; upper images) as well as low-power images of metastatic lung tissue (arrows; lower images). Scale bars: 100 µm (B), 50 µm (C), 50 µm (D, upper panel), 5000 µm (D, lower panel).

Despite the dramatic changes in pericyte association, vessel leakage and BM architecture, no significant difference in tumour growth (Fig. 3A), blood vessel density (Fig. 3B), numbers of functional blood vessels (Fig. 3C), metastasis (Fig. 3D) or immune cell infiltrate (Fig. S3B) was observed between *pdgfrβcre-;α6fl/fl* and *pdgfrβcre+;α6fl/fl* mice. These data suggest that the changes in blood vessels observed in *pdgfrβcre+;α6fl/fl* mice are not sufficient to affect tumour growth, angiogenesis or metastasis.

### α6-integrin controls pericyte PDGFRβ expression

**Fig. 4.**
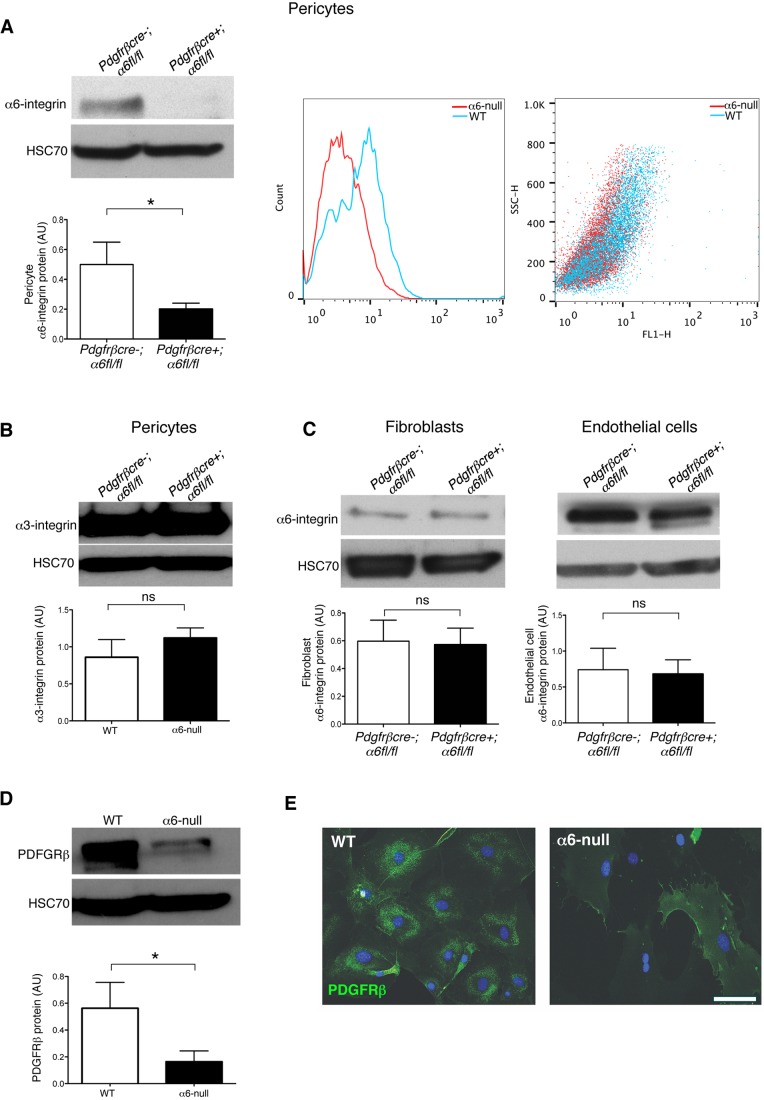
**Pericyte α6-integrin regulates PDGFRβ expression.** (A) Pericytes were isolated from *pdgfrβcre-;α6fl/fl* and *pdgfrβcre+;α6fl/fl* mice, and western blot analysis performed to assess α6-integrin deletion. α6 deletion was only observed in pericytes from *pdgfrβcre+;α6fl/fl* mice. FACS analysis also confirmed loss of α6-integrin surface levels in pericytes isolated from *pdgfrβcre+;α6fl/fl* mice compared with pericytes isolated from *pdgfrβcre-;α6fl/fl* mice (line graph and equivalent data dot plot). Bar charts represent mean+s.e.m. densitometric readings of western blots, corrected for loading. *n*=3 separate lysates/genotype. (B) Western blot analysis confirmed that wild-type (WT) and α6-null pericytes have similar levels of α3-integrin, a second laminin receptor, suggesting that there was no compensation of α3-integrin in the absence of α6-integrin. Results are mean+s.e.m.; *n*=3 separate lysates/genotype. (C) Fibroblasts and endothelial cells were isolated from *pdgfrβcre-;α6fl/fl* and *pdgfrβcre+;α6fl/fl* mice and western blot analysis performed to assess α6-integrin levels. α6-integrin levels were not affected in either cell type isolated from either *pdgfrβcre-;α6fl/fl* and *pdgfrβcre+;α6fl/fl* mice. Results are mean+ s.e.m.; *n*=3 separate lysates/genotype. (D) Western blot analysis revealed PDGFRβ protein levels were significantly reduced in primary α6-null pericytes. The bar chart shows mean+s.e.m. densitometric values of PDGFRβ levels, corrected for loading. HSC70 was used as a loading control; *n*=3 separate lysates/genotype. (E) Immunostaining of PDGFRβ in α6-null pericytes in culture was significantly reduced compared with WT pericytes. *P<0.05; ns, no significant difference. Scale bar: 50 µm.

In mice, genetic ablation of PDGF-BB and PDGFRβ leads to various vascular abnormalities associated with pericyte loss, including a loss of pericyte association with blood vessels (Abramsson et al., 2003). Since PDGF-BB is one of the major growth factors involved in pericyte investment to blood vessels (Abramsson et al., 2003; Furuhashi et al., 2004), we sought to investigate whether loss of α6-integrin affected pericyte responses to PDGF-BB. Initially, primary pericytes, fibroblasts and endothelial cells were isolated from *pdgfrβcre-;α6fl/fl* and *pdgfrβcre+;α6fl/fl* mice and characterised (Fig. S4A–C). Western blot analysis and fluorescence-activated cell sorting (FACS) confirmed deletion of α6-integrin only in pericytes (Fig. 4A). This loss of α6-integrin was not compensated for by the overexpression of another laminin receptor, the α3-integrin subunit in α6-null pericytes (Fig. 4B). Furthermore, western blot analysis showed that α6-integrin levels were normal in fibroblasts and endothelial cells isolated from *pdgfrβcre-;α6fl/fl* and *pdgfrβcre+;α6fl/fl* mice (Fig. 4C). The loss of α6-integrin in pericytes correlated with an ∼2-fold reduction in levels of PDGFRβ in α6-null pericytes, suggesting that α6-integrin was indeed regulating the expression of PDGFRβ protein (Fig. 4D). Additionally, PDGFRβ levels were significantly reduced in PDGFRβ-immunostained α6-null pericytes compared with WT pericytes *in vitro* (Fig. 4E).

### PDGF-BB responses are diminished in α6-null pericytes

**Fig. 5.**
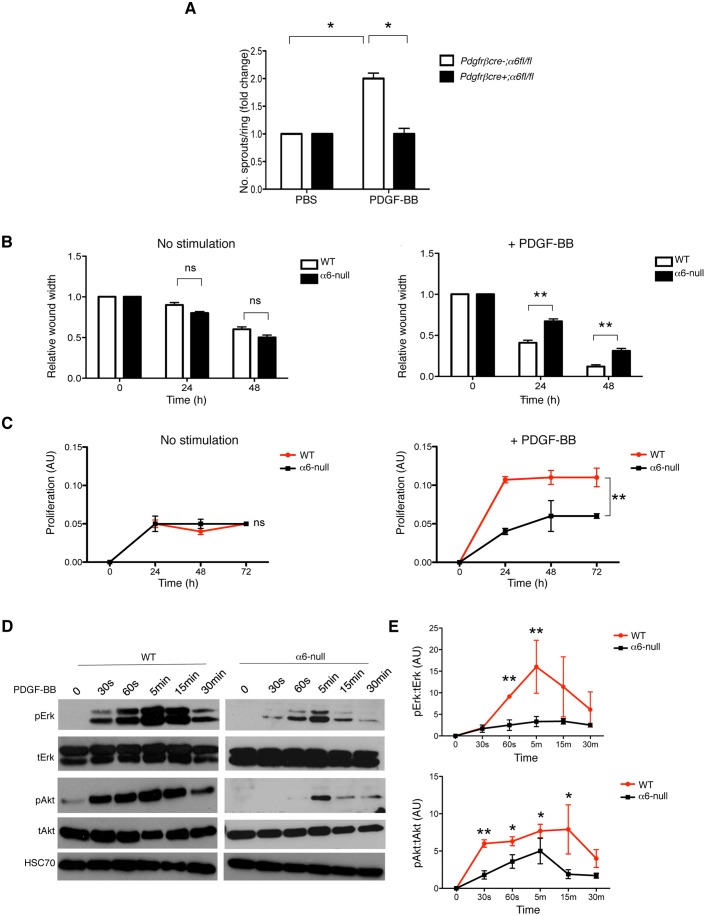
**Responses to PDGF-BB are reduced in α6-null pericytes.** (A) Aortic rings were isolated from *pdgfrβcre-;α6fl/fl* and *pdgfrβcre+;α6fl/fl* mice and treated with either PBS or PDGF-BB (30 ng/ml) for up to 10 days. PDGF-BB stimulation increased vessel sprouting in *pdgfrβcre-;α6fl/fl* but not *pdgfrβcre+;α6fl/fl* aortic rings. Bar charts show the mean+s.e.m. number of aortic sprouts/genotype; *n*=40–50 rings/genotype from triplicate experiments. (B) Confluent monolayers of primary wild-type (WT) and α6-null pericytes were wounded manually after 24 h serum starvation, then stimulated, or not, with PDGF-BB (30 ng/ml). Wound width was quantified for up to 48 h. Wound closure was significantly reduced in α6-null pericytes in response to PDGF-BB. Bar charts show mean+s.e.m. wound width in the absence (left panel) or presence (right panel) of PDGF-BB, normalised to time 0; *n*=6/genotype/time point from triplicate experiments. (C) Proliferation of primary WT and α6-null pericytes in the presence of Optimem or PDGF-BB was measured. α6-null pericytes proliferated significantly less in the presence of PDGF-BB compared with WT pericytes. Graphs represent mean±s.e.m. relative proliferation; *n*=3 biological repeats. (D) Western blot analysis of phosphorylated-ERK1/2 (pErk), total ERK1/2 (tErk), phosphorylated AKT (pAkt) and total AKT (tAkt), from WT and α6-null pericyte lysates after serum starvation and stimulation with PDGF-BB for 0, 30 s, 60 s, 5 min, 15 min, 30 min. Downstream signalling responses to PDGF-BB in α6-null pericytes were reduced significantly. Individual cropped blots are representative of samples run on the same gel under identical experimental conditions. HSC70 acted as the loading control. (E) Graphs show mean±s.e.m. densitometric readings of fold change for p-AKT to total AKT normalised to time 0, and fold change of ratios p-ERK1/2 to total ERK1/2 normalised to time 0; *n*=3 independent experiments. *P<0.05; **P<0.005; ns, no significant difference.

Since changes in receptor expression levels do not always reflect changes in downstream signalling of the corresponding tyrosine kinase receptor (Platta and Stenmark, 2011), we next sought to confirm whether the decreased expression of PDGFRβ correlated with diminished responses to PDGF-BB stimulation in α6-null pericytes. We showed that PDGF-BB-stimulated *ex vivo* microvessel sprouting was reduced in aortic rings isolated from *pdgfrβcre+;α6fl/fl* mice compared with PDGF-BB-stimulated sprouting from *pdgfrβcre-;α6fl/fl* mouse aortic rings (Fig. 5A). In this assay, the vessel sprouts become surrounded by pericytes that proliferate and migrate along the endothelium (Nicosia, 2009). We were unable to analyse pericyte coverage in *pdgfrβcre+;α6fl/fl* aortic rings due to the complete lack of sprouts that grew in response to PDGF-BB. In response to PDGF-BB, α6-null pericyte migration and proliferation was significantly reduced when compared with WT controls (Fig. 5B,C). We next examined the effect of α6-integrin deficiency on PDGF-BB-stimulated downstream signalling in pericytes. Interaction of PDGFRβ with PDGF-BB activates several signalling pathways, including the MAPK pathway (through ERK1/2, also known as MAPK3 and MAPK1) and phosphoinositide 3-kinase (PI3K) through the AKT pathway. Western blot analysis showed that, in α6-null pericytes, PDGF-BB-mediated stimulation of ERK1/2 and AKT (AKT1/2/3) pathways were both reduced significantly (Fig. 5D,E). Since integrins and PDGFR can activate many downstream signalling pathways, we performed a non-candidate proteomic study using Reverse Phase Protein Array (RPPA) analysis, to identify other possible pathways that may be affected by the absence of α6-integrin on pericytes. We found that components of the AKT–mTOR signalling pathway [AKT, P70 S6kinase (RPS6KB1), mTOR, pS6 ribosomal protein (RPS6) and 4E-BP1] were significantly downregulated in α6-null pericytes (Fig. S4D). This pathway is activated downstream of integrins and is known to be regulated by PDGFR expression and function (Zhang et al., 2007). These results suggest that absence of α6-integrin results in a reduction of PDGFRβ levels, significantly reducing pericyte responses to PDGF-BB.

Collectively, these results demonstrate a novel role for pericyte α6-integrin in both the regulation of PDGFRβ levels, which was previously unknown, and in BM organisation. This dual mechanism confers a blood vessel phenotype that affects only the primary tumour.

## DISCUSSION

The role of pericyte α6-integrin expression has not been addressed previously. We have now shown that the combined effects of the downregulation of PDGFRβ and the changes in pericyte adhesion upon deletion of α6β1-integrin induces destabilisation of tumour blood vessels. Genetic ablation of pericyte α6β1-integrin correlates with reduced pericyte investment to tumour blood vessels, changes in BM architecture and reduced PDGFRβ expression levels and PDGF-BB-mediated downstream signalling. These correspond with increased blood vessel leakage without affecting tumour growth or metastasis. Taken together, our results suggest a dual function of α6-integrin on pericytes, and here we will discuss both the effect of reduced PDGFRβ levels and decreased pericyte adhesion in turn.

Very few studies have linked pericyte integrins with growth factor receptor regulation. Currently, α5β1-integrin has been shown to regulate signalling through PDGFRβ in vascular smooth muscle cells (Veevers-Lowe et al., 2011), and inactivation of PDGF-BB signalling can decrease α1β1 integrin levels (Hosaka et al., 2013). In another study, NG2 depletion in pericytes was shown to reduce β1-integrin-mediated signalling (You et al., 2014). Although limited, these studies suggest possible cross-talk between pericyte integrins and growth factor receptors; a mechanism that has been shown previously between endothelial cell integrins and growth factor receptors (da Silva et al., 2010; Germain et al., 2010; Reynolds et al., 2002). In parallel, *in vitro* studies using fibroblasts have highlighted the ability of integrins to enhance PDGF-dependent responses (DeMali et al., 1999; Sundberg and Rubin, 1996). Here, we show for the first time that α6β1-integrin acts as a regulator of PDGFRβ – controlling its expression and signalling upon stimulation with PDGF-BB – in pericytes. Our data indicate that depletion of α6-integrin on pericytes leads to a significant reduction in the levels of PDGFRβ. In turn, this leads to a downregulation in the activation of the MAPK and AKT signalling pathways, which are both known to be critical for cell migration and proliferation (Lemmon and Schlessinger, 2010). Our observation of reduced pericyte PDGFRβ levels, signalling and resulting inhibited responses to PDGF-BB in α6-integrin deficient pericytes may explain the tumour blood vessel phenotypes we observe in the *pdgfrβcre+;α6fl/fl* mice, including reduced pericyte blood vessel investment and increased detachment of pericytes to endothelial cells, since these functions have been reported to be mediated by PDGF-BB (Abramsson et al., 2003; Hellberg et al., 2010). It has been well documented that tumours transplanted into PDGF-B retention motif-deficient (*pdgf-b^ret/ret^*) mice have an ∼50% reduction in numbers of pericytes that associate poorly with the blood vessel wall and result in leaky vessels (Abramsson et al., 2003). It is noteworthy that although we observe a similar leaky blood vessel phenotype to that reported in tumour growth in *pdgf-b^ret/ret^* mice, our results indicate that α6β1-integrin is a regulator of pericyte function rather than numbers, as opposed to the reduction in numbers seen in the *pdgf-b^ret/ret^* mice (Lindblom et al., 2003).

One surprising observation was that despite such striking blood vessel defects in the *pdgfrβcre+;α6fl/fl* mouse tumours, including increased vessel leakage, we did not observe any changes in tumour growth, angiogenesis or lung metastasis (Cooke et al., 2012; Zang et al., 2015). Our work is in line with previous studies showing that early ablation of NG2+ cells or depletion of PDGFβR+ pericytes does not necessarily affect tumour growth and metastasis (Keskin et al., 2015) suggesting that increased leakage alone is not sufficient to enhance metastasis. Indeed, increased vascular leakage has been shown to be insufficient for metastasis per se (Thurston et al., 1999). We hypothesise that despite the observed decrease in blood vessel pericyte coverage in *pdgfrβcre+;α6fl/fl mice*, the remaining pericytes attached to the endothelial cells provide enough survival factors, for example VEGF and Ang-1 (angiopoietin-1, also known as Angpt1), to allow endothelial cells to survive, allowing for normal tumour angiogenesis and tumour growth. Studies have shown that decreased pericyte coverage can lead to regression of blood vessels but the magnitude is tumour specific and does not necessarily retard tumour growth (Sennino et al., 2007). Indeed, treatment of RipTag2 tumours with anti-PDGFRβ antibody reduces pericyte numbers and enlarges blood vessels but does not reduce tumour vascular density (Song et al., 2005). Although not within the scope of this study, we believe that the phenotype observed in our *pdgfrβcre+;α6fl/fl* mice may help to improve chemotherapy efficacy for primary tumours owing to the leaky vessel defect.

As well as pericyte α6-integrin deficiency affecting PDGFRβ levels and upstream signalling, we also show that deletion of α6-integrin affects the ECM adhesion properties of pericytes *in vitro* and *in vivo*. The BM that surrounds blood vessels is necessary for vessel integrity, stability and maturation. Recent studies have highlighted the importance of normal endothelial cell–pericyte interactions for proper BM organisation (Davis et al., 2013; Stratman et al., 2009; Baluk et al., 2003). When this interaction is de-stabilised, BM organisation is affected, resulting in decreased vessel integrity (Stratman et al., 2009; Davis et al., 2013). Therefore, it is possible that the phenotype we observe in the microvessel BM of *pdgfrβcre+;α6fl/fl* mice may be due, at least in part, to a loss of pericyte adhesion to the endothelial cell basement membrane. Similar phenotypes have been shown in other studies; for example, inactivation of the β1-integrin subunit in mural cells leads to failure of these cells to associate with the subendothelial BM (Abraham et al., 2008), and genetic ablation of a related laminin receptor, α3-integrin, results in an epidermal BM defect in which components of the BM show a disorganised expression pattern (DiPersio et al., 1997; Georges-Labouesse et al., 1996; Has et al., 2012) very similar to that observed in tumour blood vessels of mice deficient in α6-integrin pericyte. Foxf2 (a forkhead transcription factor specifically expressed by pericytes) deficiency in brain pericytes leads to significantly reduced PDGFRβ and αvβ8-integrin levels with thinning of the vascular basal lamina, resulting in a leaky blood–brain barrier (Reyahi et al., 2015); inactivation of PDGF-BB signalling decreases α1β1-integrin levels and impairs pericyte adhesion to ECM components of blood vessels (Hosaka et al., 2013).

Taken together, these studies all support a common notion that crosstalk between pericyte integrins with PDGFRβ signalling can affect vascular BM organisation and vessel function. It is also conceivable that microvessel BM disorganisation may be a contributing cause to decreased supporting cell coverage in the tumour blood vessels in *pdgfrβcre+;α6fl/fl* mice. For example, it has been shown that deletion of laminin-α4 chain in mice causes impaired vessel growth due to reduced pericyte recruitment to blood vessels (Abrass et al., 2010). *In vitro*, pericyte recruitment during tube formation is necessary to stimulate endothelial BM formation (Stratman et al., 2009). Overall, our data suggest that the absence of pericyte α6-integrin leads to (1) a reduced investment of pericytes to tumour microvessels possibly due to reduced PDGFRβ levels, and (2) that this is associated with poor vessel BM architecture leading to vascular leakage.

Our study provides new insights into the regulation of tumour blood vessels by pericyte α6-integrin, which points towards an important role in the regulation of tumour vessel leakage.

## MATERIALS AND METHODS

### Generation of mice

α6-integrin floxed mice (Bouvard et al., 2012; Germain et al., 2010) were bred with mice expressing Cre-recombinase under the control of the PDGFRβ promoter, PDGFRβCre (Foo et al., 2006), to generate *pdgfrβcre-;α6fl/fl* and *pdgfrβcre+;α6fl/fl* mice.

### Immunostaining of tumour sections

Unless otherwise stated, frozen sections were fixed in ice-cold acetone for 10 min followed by permeabilisation with 0.5% NP-40 for 10 min. Sections were blocked for 45 min with 1.0% bovine serum albumin (BSA) and 0.1% Tween 20 in PBS. Primary antibodies were incubated overnight at 4°C followed by incubation with a fluorescently conjugated secondary antibody for 1 h at room temperature (1:1000; Invitrogen). Primary antibodies against the following were used: α6-integrin (GoH3, Chemicon), NG2 (AB5320, Millipore), endomucin (sc-65495, Santa Cruz Biotechnology) (all 1:100).

### Quantification of α6-integrin on pericytes

Quantification of α6-integrin expression on NG2-positive pericytes on tumour blood vessels from *pdgfrβcre-;α6fl/fl* and *pdgfrβcre+;α6fl/fl* mice was performed using ImageJ software. The mean pixel intensity of α6-integrin expression on NG2-positive pericytes was quantified.

### Immunostaining and quantification of BM

Immunostaining for laminin α4 chain (antibody was a kind gift from Takako Sasaki, Dept. Matrix Medicine, Oita University, Japan) was performed as described previously (Sasaki et al., 2001). For laminin α5 chain, sections were fixed in 4% paraformaldehyde (PFA), washed twice with PBS, then blocked with 1% BSA for 30 min. Sections were incubated overnight at 4°C with primary antibody against laminin α5 (1:400 dilution in blocking buffer; kind gift from Jeffrey H. Miner, Division of Biology & Biomedical Sciences, Washington University in St Louis, USA; Pierce et al., 2000), followed by several washes, incubation with Alexa-Fluor-488-conjugated anti-rabbit-IgG secondary antibody (1:1000; Invitrogen) and mounted.

For collagen IV (ab19808, Abcam) and fibronectin (ab23750, Abcam) staining, sections were fixed in 4% PFA, blocked with 3% normal goat serum (NGS), 0.1% Triton X-100 (TX-100) in PBS for 30 min at room temperature. Primary antibody was diluted 1:200 in 1% NGS, 0.1% TX-100 in PBS and incubated overnight at 4°C. Sections were washed three times with PBS, incubated with Alexa-Fluor-488-conjugated anti-rabbit secondary antibody (Invitrogen) diluted 1:100 in 1% NGS TX-100, washed three times and mounted with Prolong Gold anti-fade with DAPI.

Fluorescence staining was visualised using the Axioplan microscope (Zeiss). Images were captured using Axiovision Rel. 4.0 software. The Axiovision software linear measuring tool was used to analyse the spread (in µm) of BM surrounding blood vessels.

### Pericyte association

For analysis of pericyte coverage, tumour sections were double immunostained for endomucin and NG2 (for details see ‘Immunostaining of tumour sections’). Pericyte coverage was quantified by counting the total number of endomucin-positive blood vessels across whole tumour sections followed by the numbers of blood vessels positive for both endomucin and NG2. The percentage of blood vessels with associated pericytes was calculated.

### Tumour blood vessel leakage

To analyse tumour blood vessel leakage, Hoechst 33258 dye (4 µg/ml; Sigma, H33258) was used. Hoechst dyes diffuse quickly from vessels and bind to the DNA of cells surrounding blood vessels, allowing for quantification of areas of uptake by perivascular tumour cells and hence blood vessel leakage (Janssen et al., 2005). Briefly, mice were injected sequentially with 100 μl phycoerythrin (PE)-conjugated CD31 (Biolegend; to stain functional blood vessels) followed 9 min later with 100 μl Hoechst 33258 (4 µg/ml; Sigma, H33258) via the tail vein and were killed 1 min later. Tumours were excised and snap-frozen. A total of 8–10 fields at ×20 magnification were analysed by ImageJ. For quantification, blood vessel leakage was calculated by counting the numbers of Hoechst 33258-positive nuclei surrounding PE–CD31-positive tumour areas. Results are shown in arbitrary units (AU).

### Tumour growth and angiogenesis

The syngeneic mouse tumour cell lines B16F0 (melanoma, derived from C57BL6) and Lewis Lung Carcinoma (LLC) (both from the ATCC) were used in subcutaneous tumour growth experiments. 1×10^6^ B16F0 cells or 0.5×10^6^ LLC cells, resuspended in 100 µl of phosphate-buffered saline (PBS), were injected subcutaneously into the flank of 12–14-week-old *pdgfrβcre+;α6fl/fl* and littermate control mice (*pdgfrβcre-;α6fl/fl* mice). Tumour growth was measured every 2 days using calipers. After 14 days, animals were culled, tumours excised and either fixed in 4% formaldehyde in PBS overnight, or snap-frozen in liquid nitrogen, for subsequent immunohistochemical analysis.

### Blood vessel density

Size-matched tumours from *pdgfrβcre-;α6fl/fl* and *pdgfrβcre+;α6fl/fl* mice were snap-frozen and bisected, and cryosections made. Frozen sections were fixed in 100% acetone at −20°C, rehydrated in PBS for 10 min, and then blocked (PBS, 1% BSA, 0.1% Tween-20) for 45 min at room temperature. After a 5 min wash in PBS, sections were incubated with 1:100 anti-endomucin (as above) in blocking buffer for 45 min at room temperature. After three 5 min washes in PBS, sections were incubated with Alexa-Fluor-488-conjugated anti-rat-IgG secondary antibody (Invitrogen) diluted in blocking buffer. After three 5 min washes in PBS, sections were washed briefly with distilled water before being mounted. The number of endomucin-positive blood vessels present across the entire area of each mid-line tumour section from size and age-matched tumours was counted and divided by the area of the section to determine tumour blood vessel density.

### Tumour blood vessel diameter

The diameter of endomucin-positive blood vessels were quantified by using the Axiovision software linear measuring tool.

### Perfused blood vessel analysis

For analysis of the percentage of perfused, functional tumour vessels, 100 μl PE–CD31 antibody (Biolegend, London, UK) was injected via the tail vein 10 min (mins) prior to killing of mice. Tumours were dissected immediately, snap-frozen and sectioned. Frozen sections were then immunostained for endomucin as described above. To calculate the percentage of functional vessels, the number of PE–CD31-positive blood vessels was divided by the total number of endomucin-positive blood vessels.

### Tumour metastasis

0.5×10^6^ LLC tumour cells were injected subcutaneously and tumours were allowed to reach a size of 100 mm^3^ before surgical resection. The mice were monitored for up to 3 weeks, after which the metastatic burden was quantified by counting numbers of surface lung metastases. Lungs were then fixed and sections stained with H&E for further analysis. The numbers of surface metastases was counted immediately after fixation, to give the total number of lung metastases per mouse. To examine internal metastases, H&E-stained sections were analysed and the area of individual metastases was measured using Axiovision software, to give the internal metastatic nodule area.

### Primary endothelial cell, fibroblast and pericyte isolation

Primary mouse endothelial cells were isolated from lungs and maintained as described previously (Reynolds and Hodivala-Dilke, 2006). Briefly, *pdgfrβcre-;α6fl/fl* and *pdgfrβcre+;α6fl/fl* mouse lungs were minced, collagenase digested (Type I, Gibco), strained through a 70 µm cell strainer (BD Falcon) and the resulting cell suspension plated on flasks coated with a mixture of 0.1% gelatin (Sigma), 10 µg/ml fibronectin (Millipore) and 30 µg/ml rat tail collagen (Sigma). Endothelial cells were purified by a single negative (FCγ sort-RII/III; Pharmingen) and two positive cell sorts (ICAM-2; Pharmingen), using anti-rat IgG-conjugated magnetic beads (Dynal). During preparation of primary endothelial cells, lung fibroblasts were isolated from the non-endothelial cell population that was generated during the first positive sort. For all cell types, passaging occurred when cells reached 70% confluency. Cells were trypsinised, centrifuged, washed with PBS and replated on pre-coated flasks for endothelial cells and pericytes and non-coated flasks for fibroblasts. Fibroblasts were cultured in Dulbecco's modified Eagle's medium (DMEM) with 10% fetal calf serum (FCS) to passage 4, endothelial cells in MLEC [Ham's F-12, DMEM (low glucose), 10% FCS, heparin and endothelial mitogen (Generon)] to passage 4–5. Pericytes were isolated from mouse brains as described previously (Tigges et al., 2012) and cultured in Pericyte medium (ScienCell) to passage 9.

### FACS analysis

Primary mouse brain pericytes isolated from *pdgfrβcre-;α6fl/fl* and *pdgfrβcre+;α6fl/fl* mice were incubated with an anti-α6-integrin antibody (1:100, GoH3; Abcam), to determine expression levels, for 30 min at 4°C. This was followed by incubation, for 30 min at 4°C with an appropriate FITC-conjugated secondary antibody. Unstained cells were used as a control. For characterisation of primary mouse brain pericytes, cells were washed with PBS and trypsinised at 37°C. The cell suspensions were washed in medium containing serum and centrifuged at 214 ***g*** for 3 min. Cells were washed with cold FACS buffer (1% BSA in PBS) and fixed with 4% formaldehyde for 10 min at room temperature. Cells were washed with FACS buffer and the cell suspensions were incubated with the following primary antibodies (all 1:100) for 30 min: PE-conjugated anti-CD31 (102507, Biolegend), PE-conjugated anti-Mac1 (CD11b; 101207, Biolegend), PE-conjugated anti-GFAP (561483, BD Biosciences), APC-conjugated anti-PDGFRβ (136007, Biolegend), PE–Cy7-conjugated anti-CD146 (134713, Biolegend). Cells were then washed three times in sample buffer and resuspended in a final volume of 400 ml. As a control, unstained cells were sorted by FACS. Primary mouse lung endothelial cells were incubated with PE-conjugated anti-CD31 (as above).

### Western blot analysis

Primary lung endothelial cells, lung fibroblasts and brain pericytes isolated from *pdgfrβcre-;α6fl/fl* and *pdgfrβcre+;α6fl/fl* mice were grown to 70–80% confluency then lysed in RIPA buffer. 15–30 µg protein was run on 8% polyacrylamide gels then transferred to nitrocellulose membranes. Membranes were probed with primary antibody overnight at 4°C. All antibodies (against ERK1/2, cat. no 9102; phosphoERK1/2, cat. no 9101; AKT, cat. no 9272; phosphoAKT, cat. no 4058; α6-integrin, cat. no 3750) for the signalling studies were purchased from Cell Signaling and used at a 1:1000 dilution. α3-integrin antibody was purchased from Millipore (AB1920, 1:1000). The anti-HSC70 antibody, used as a loading control, was from Santa Cruz Biotechnology (cat. no sc-7298) and was used at 1:5000 dilution. For PDGF-BB stimulation, pericytes were serum starved for 6 h in Optimem with 0.5% FCS, then stimulated with PDGF-BB (30 ng/ml; Peprotech, UK) for 0, 30 s, 60 s, 5 min, 15 min and 30 min before lysis. Densitometric readings of band intensities were obtained using the ImageJ software.

### PDGFRβ immunostaining of cells

Immunostaining of brain pericytes, isolated from *pdgfrβcre-;α6fl/fl* and *pdgfrβcre+;α6fl/fl* mice for PDGFRβ (1:1000, 28E1, Cell Signaling), was performed according to the manufacturer's protocol.

### Aortic ring assay

Thoracic aortas were isolated from *pdgfrβcre-;α6fl/fl* and *pdgfrβcre+;α6fl/fl* 8–10-week-old mice and prepared for culture as described previously (Baker et al., 2012). Where indicated, culture medium was supplemented with PDGF-BB at 30 ng/ml (Peprotech, London, UK). PBS was used as a control. Aortic rings were fed every 3 days with fresh medium with or without PDFG-BB (30 ng/ml). Sprouting microvessels were counted after 9 days in culture, fixed and stained to identify endothelial cells and pericytes, as described previously (Baker et al., 2012).

### Scratch wound assays

Primary mouse brain pericytes from *pdgfrβcre-;α6fl/fl* and *pdgfrβcre+;α6fl/fl* mice were grown to confluency in Pericyte medium (ScienCell, cat no. 0010) in six-well plates coated with 0.1% gelatin and fibronectin. Cells were serum starved overnight in Optimem containing 0.5% FCS. The following day, the monolayers were scratched horizontally and vertically through the centre of each well. The cells were either stimulated with PDGF-BB (30 ng/ml) or not and cell migration monitored over a 72 h period. At each time point, 12 photographs were taken of each scratch, and the wound width was measured using ImageJ software. Results were normalised to the wound width at time 0.

### Proliferation assay

Primary WT and α6-null brain pericyte proliferation was assessed using the CellTiter 96^®^ Aqueous One Solution Reagent (Promega), according to the manufacturer's instructions. Plates were read at a wavelength of 490 nm, with absorbance measured relative to blank wells containing reagent only. Plates were coated with 0.1% gelatin and fibronectin prior to seeding the pericytes.

### Characterising transgenic mice

The primers for the Cre PCR were forward primer, 5′-GCCGCATTACCGGTCGATGCAAGA-3′ and reverse primer, 5′-GTGGCAGATGGCGCGGCAACACCATT-3′. The reaction generates a fragment of ∼1000 bp. The primers for α6-floxed PCR were forward primer, 5′-AGAAGGTGATGTTACCCT-3′ and reverse primer, 5′-AATGTAACTAGCATTCAAGT-3′. The PCR generates a 154 bp fragment for the α6-ﬂoxed allele and a 120 bp fragment for the wild-type allele (described in Germain et al., 2010).

### Whole-mount immunofluorescence on retinas

Postnatal day (P)9 eyes were fixed in 4% PFA overnight at 4°C. Retinas were dissected in PBS and, after five washes in PBS, incubated in blocking solution [1% FBS (Sigma), 3% BSA (Sigma), 0.5% Triton X-100 (Sigma), 0.01% Na deoxycholate (Sigma), 0,02% Na Azide (Sigma) in PBS, pH 7.4] for 2 h at room temperature. Retinas were incubated overnight at 4°C with rabbit polyclonal anti-NG2 (Millipore, #AB5320, dilution 1:500) in blocking solution:PBS (1:1) at 4°C. Retinas were then washed several times in PBS and incubated with secondary antibody conjugated to Alexa Fluor 488 and 548 (Life Technologies), all diluted 1:300, overnight at 4°C. After several washes in PBS, retinas were post-fixed in 1% PFA and re-blocked in 1% BSA and 0.5% Triton X-100 in PBS for 1 h at room temperature. After two rinses in PBlec (0.1 nM CaCl_2_, 0.1 mM MgCl_2_ , 0.1 mM MnCl_2_ and 1% Triton X-100 in PBS, pH 6.8), retinas were incubated with biotinylated Isolectin B4 (B1205, VectorLabs, dilution 1:12.5) in PBlec overnight at 4°C. After several washes in PBS, retinas were incubated with Alexa Fluor 647-conjugated Streptavidin diluted 1:100 in 0.5% BSA with 0.3% Triton X-100 in PBS overnight at 4°C. After several washes in PBS, retinas were incubated with Hoechst 33342 dye as a nuclear counterstain (H3570, Life Technologies), washed and mounted with ProLong Gold (Molecular Probes). Fluorescently labelled samples were imaged with a confocal microscope (Carl Zeiss LSM 710, Carl Zeiss) in multichannel mode. Three-dimensional projections were digitally reconstructed from confocal *z*-stacks using the LSM Zen 2009 software and Image J open source image processing software (version:2.0.0-rc-43/1.51d).

### Adhesion assay

Adhesion assays were performed as previously described (Germain et al., 2010), using brain pericytes isolated from *pdgfrβcre-;α6fl/fl* and *pdgfrβcre+;α6fl/fl* mice. Adhesion of pericytes is presented relative to adhesion of pericytes to fibronectin for the same genotype.

### Immunostaining of immune cells, fibroblasts and endothelial cells

Immunostaining of immune cells from B16F0 tumour sections was performed as described previously (Reynolds et al., 2010).

Primary mouse lung fibroblasts and endothelial cells were fixed with 4% PFA for 10 min, washed twice and blocked with 5% NGS in PBS for 30 min at room temperature. Primary antibodies to vimentin (1:100; 5741, Cell Signaling), NG2 (1:100; MAB 5385, Millipore), endomucin (1:100; V7C7, Santa Cruz Biotechnology) and α-Sma (clone 1A4, Sigma) were incubated for 1 h at room temperature, washed three times with PBS, and incubated with the relevant secondary antibody for 45 min at room temperature.

### RPPA nitrocellulose slides

Cell lysates were prepared using ice-cold RIPA buffer. After normalisation, to adjust protein concentrations, triplicate spots of each lysate were deposited onto 16-pad Avid Nitrocellulose slides (Grace Bio) under conditions of constant 70% humidity using an Aushon 2470 Array platform (Aushon BioSystems). After printing and washing steps, the arrays were blocked by incubation in Superblock (Thermo Scientific #37535) for 10 min. The protein array chips were subsequently incubated for 1 h with primary antibody followed by repeat blocking with Superblock and 30 min incubation with anti-rabbit Dylight-800-conjugated secondary antibody (Cell Signaling, cat. no. 5151).

Following secondary antibody incubation and subsequent wash steps, the immune-stained arrays were imaged using an Innopsys 710IR scanner (Innopsys, France). Microarray images were obtained at the highest gain without saturation of fluorescent signal detection. Image analysis was performed using Mapix software (Innopsys, France) to calculate the relative fluorescence intensity (RFI) value for each sample. An estimate of total protein printed per feature on the array was determined by staining an array slide with fastgreen protein stain. Readout values for all antibodies tested are expressed as a ratio of the total protein loaded and are presented as the mean of technical replicates.

### Ethical regulations

All animals were used in accord with United Kingdom Home Office regulations (Home Office license number 70/7449). The in-house Ethics Committee at Queen Mary University of London has approved all experiments, using mice under the project license.

### Statistical analysis

Statistical significance was calculated by using a Student's *t*-test. *P*<0.05 was considered statistically significant.
